# Analysis of a miR-148a Targetome in B Cell Central Tolerance

**DOI:** 10.3389/fimmu.2022.861655

**Published:** 2022-05-12

**Authors:** Fengge Ma, Yating Zhan, Rocío Bartolomé-Cabrero, Wei Ying, Masahide Asano, Zhe Huang, Changchun Xiao, Alicia González-Martín

**Affiliations:** ^1^State Key Laboratory of Cellular Stress Biology, Innovation Center for Cell Signaling Network, School of Life Sciences, Xiamen University, Xiamen, China; ^2^Department of Biochemistry, Universidad Autónoma de Madrid (UAM), Instituto de Investigaciones Biomédicas Alberto Sols (CSIC-UAM), Madrid, Spain; ^3^Institute of Laboratory Animals, Graduate School of Medicine, Kyoto University, Kyoto, Japan; ^4^Department of Immunology and Microbiology, The Scripps Research Institute, La Jolla, CA, United States

**Keywords:** B cell tolerance, microRNA, clonal deletion, miR-148a, target gene

## Abstract

A microRNA (miRNA) often regulates the expression of hundreds of target genes. A fundamental question in the field of miRNA research is whether a miRNA exerts its biological function through regulating a small number of key targets or through small changes in the expression of hundreds of target genes. We addressed this issue by performing functional analysis of target genes regulated by miR-148a. We previously identified miR-148a as a critical regulator of B cell central tolerance and found 119 target genes that may mediate its function. We selected 4 of them for validation and demonstrated a regulatory role for *Bim*, *Pten*, and *Gadd45a* in this process. In this study, we performed functional analysis of the other miR-148a target genes in *in vitro* and *in vivo* models of B cell central tolerance. Our results show that those additional target genes play a minimal role, if any, in miR-148a-mediated control of B cell central tolerance, suggesting that the function of miRNAs is mediated by a few key target genes. These findings have advanced our understanding of molecular mechanisms underlying miRNA regulation of gene expression and B cell central tolerance.

## Introduction

B cells mount humoral responses against pathogens following their recognition of a vast variety of foreign antigens through their B cell receptors (BCRs). The large diversity of BCR specificities required for this is generated through V(D)J recombination of the heavy and light chains of the immunoglobulin (Ig) M during the early pro-B and pre-B stages of B cell development in the bone marrow. The largely random and combinatorial nature of these rearrangements also generates many autoreactive B cells that recognize self-tissues and, thus, can lead to autoimmunity. To test their autoreactivity, IgM-expressing immature B cells are scrutinized by the process of central tolerance. If a B cell is autoreactive, it continues to rearrange its light chain to eliminate self-reactivity through receptor editing. If this process is not successful, the autoreactive cell is deleted from the repertoire by apoptosis. This is known as clonal deletion. If the cell is non-autoreactive or eliminated its autoreactivity through receptor editing, it exits to the periphery to become a mature B cell (1–3). B cell central tolerance is a tightly regulated process, but the molecular mechanisms governing this checkpoint remain largely unexplored. Expanding this knowledge is important because defects in B cell tolerance have been shown in many patients with autoimmune diseases such as systemic lupus erythematosus (SLE), type 1 diabetes, rheumatoid arthritis, and multiple sclerosis (4, 5).

MiRNAs are small non-coding RNAs of approximately 22 nucleotides in length that regulate the expression of protein-coding genes at the post-transcriptional level, resulting in a fast and robust control of virtually all cellular processes, including development, activation, proliferation, differentiation, and apoptosis (6–12). MiRNA expression levels are tightly regulated and highly conserved throughout evolution, which supports their functional importance (13–15). Hundreds of genes can be targeted in any given cell by an individual miRNA through its cognate binding sites on their mRNAs (16, 17). MiRNA studies frequently validate a limited number of predicted or experimentally identified target genes, without examining all potential target genes in a systematic manner. Hence, a fundamental question in the field that remains to be answered is whether miRNAs exert their function in a given cellular context through simultaneous regulation of a large number of target genes or, alternatively, through a few critical ones (11, 16, 18, 19).

In a recent study, we identified miR-148a as the first miRNA that functions as a critical regulator of B cell tolerance using the IgM^b^-macroself mouse model of B cell tolerance (20). The IgM^b^-macroself mice ubiquitously express an engineered membrane-bound superantigen reactive to the constant region of IgM heavy chain. In these mice, early B cell development occurs normally, resulting in an immature B cell pool with a large variety of antigen specificities in their bone marrow. However, once immature B cells express IgM molecules on the cell surface, they are recognized by the engineered superantigen. This mimics the recognition of self-antigens by BCR. These “autoreactive” B cells first attempt to eliminate their autoreactivity through receptor editing and then die by apoptosis (clonal deletion), resulting in the absence of B cells in the peripheral lymphoid organs. This murine model provides a robust and clean system to identify novel regulators of B cell tolerance through a hematopoietic and precursor stem cell (HPSC) transduction and reconstitution approach or reconstitution with bone marrow cells from transgenic or knockout mice, with the presence of B cells in the spleens of reconstituted IgM^b^-macroself mice indicating a break of B cell tolerance. MiR-148a expression is tightly regulated during B cell development and differentiation, and its expression levels frequently increased in lymphocytes of patients with lupus erythematosus and lupus-prone mice even before disease onset (13, 20–22). Increased expression of miR-148a in B cells compromised B cell tolerance in IgM^b^-macroself mice and facilitated lethal autoimmunity in MRL-lpr mice, a classical mouse model of lupus. Importantly, this effect was recapitulated in WEHI-231 cells, an *in vitro* model of clonal deletion. Moreover, 119 protein coding genes were identified as potential miR-148a target genes. Four of them were further investigated and 3 of them (*Bcl2l11*, *Pten* and *Gadd45a)* were demonstrated to be bona fide miR-148a target genes and regulate B cell central tolerance in IgM^b^-macroself mice (20).

However, this study did not investigate the potential contribution of the other 115 target genes to miR-148a regulation of B cell tolerance. This provided the opportunity to perform a systematic and unbiased functional analysis of a miRNA targetome in B cell tolerance to determine whether this miRNA exerts its biological function through a large number or only a few key target genes in this cellular context. No study addressing this question has been previously conducted to our knowledge. By performing *in vitro* and *in vivo* functional analysis of miR-148a target genes in B cell tolerance, this work aims to advance our current understanding of miRNA biology in B cell tolerance, with potential implications for the therapy of autoimmune diseases.

## Materials and Methods

### Mice

*B4galt5*^fl/+^ mice were obtained from Masahide Asano (23). *B4galt5*^fl/+^ mice were bred with Mb1Cre mice (The Jackson Laboratory, Stock 020505) to obtain Mb1Cre;*B4galt5*^fl/fl^ mice. *Nsd1*^fl/+^ mice were purchased from Gempharmatech (T018412), bred with Mb1Cre mice to obtain Mb1Cre;*Nsd1*^fl/fl^ mice. IgM^b^-macroself mice were generated by David Nemazee´s laboratory (The Scripps Research Institute, La Jolla, CA) as previously described (20, 24). All mouse strains were housed and bred in pathogen-free conditions.

### Cell Culture

The immature B cell line WEHI-231 was originally obtained from David Nemazee (The Scripps Research Institute, La Jolla, CA) and cultured in DMEM (Gibco, Cat:10569-044, containing GlutaMAX and Sodium Pyruvate) supplemented with 10% FBS, 1% Pen/Strep, and 50 μM 2-mercaptoethanol. The HEK293T cell line was originally obtained from Jun Lu (Yale University, New Haven, CT) and cultured in DMEM (Gibco, Cat:10569-044, containing GlutaMAX and Sodium Pyruvate) supplemented with 10% FBS and 1% Pen/Strep.

### Retroviral TransOMIC-shRNA Library Generation

The TransOMIC-shRNA sublibrary was obtained from the RNAi Core at La Jolla Institute for Allergy and Immunology (La Jolla, CA), amplified from a glycerol stock, and DNA prepared using an endotoxin-free DNA purification kit (K210001, ThermoFisher). Retrovirus preparations were obtained as previously described (20). Briefly, packaging vectors GAG-POL, VSV-G and individual shRNA-encoding or empty plasmids were co-transfected into HEK293T cells using TransIT-LT1 (Mirus) following the manufacturer´s instructions. Supernatants containing retrovirus were collected after 48 and 72 hours, filtered through a 0.45μm low protein retention filter, aliquoted and stored at −80°C until use.

### Flow Cytometry Analysis

For WEHI-231 apoptosis analysis, the active Caspase 3 kit (C92-605, BD Pharmingen) was used following the manufacturer’s instructions. Briefly, cells were fixed and permeabilized with Fix/Perm Buffer for 30 min at 4°C, stained with an antibody for active Caspase 3 labelled with APC for 30 min at 4°C, and analyzed by flow cytometry. For splenic B cell analysis of IgM^b^-macroself mice reconstituted with retrovirus-transduced HPSCs or bone marrow cells from donor mice, single-cell suspensions were obtained by disaggregating spleens through a 0.70μm cell strainer, depleted of red blood cells by incubation for 5 min RT with ACK lysis buffer, stained with CD45.1-PerCP/Cy5.5 (A20; Biolegend), CD45.2-PE (104; Biolegend), IgM-APC (RMM-1; Biolegend) and CD19 PE/Cy7 (1D3, eBioscience) for 20 min at 4°C, and analyzed by flow cytometry. Data were acquired on FACS Calibur, Novocyte or Fortessa X-20 flow cytometers, and analyzed with Flowjo Version10.7.1.

### *In Vitro* Screen of Stable shRNA WEHI- 231 Cell Lines

To generate stable WEHI-231 cell lines, 0.5x10^6^ cells were transduced with retroviruses encoding individual shRNAs in 24-well plates overnight. Media was replaced with fresh one the next morning. After 24 hours of culture, 10µg/ml puromycin was added to medium to select transduced cells for 5 days. After selection, stable cell lines were maintained in the presence of 2µg/ml puromycin in culture medium. Stable WEHI-231 cells were stimulated with 2µg/ml anti-IgM (115-006-020, µ chain specific, Jackson) for 72 hours, stained for intracellular active caspase-3, and analyzed by flow cytometry as described above.

### *In Vivo* Validation of Positive Hits in IgM^b^-Macroself Mice

For *in vivo* validation, plasmids encoding positive shRNA hits were modified to delete the puromycin resistance gene, amplified using an endotoxin-free DNA purification kit (12362, Qiagen), and packaged to generate retroviral supernatants as described above. For HPSC enrichment, wild type C57BL/6J mice were treated with 4 mg/mouse 5-fluorouracil (5-FU) (Sigma) by intravenous (iv) injection, followed by bone marrow cell collection 5 days later. HPSCs were stimulated with SCF (100ng/ml, Novoprotein), IL-3 (20ng/ml, Novoprotein) and IL-6 (25ng/ml, Novoprotein) for 36 hours before transduction. Retrovirus transduction of HPSCs was performed by spinoculation (2000rpm, 2 hours without brake) and media was replaced 4 hours later. Transduced cells were harvested after 20 hours for intravenous transfer into IgM^b^-macroself mice. Alternatively, freshly isolated BM cells from mutant mice were depleted of red blood cells using ACK lysis buffer, counted, and resuspended in PBS. 1-10x10^6^ retrovirally transduced HPSCs or freshly isolated BM cells were iv injected into recipient mice. IgM^b^-macroself mice were irradiated with 6 Gy by X-Ray before reconstitution. Recipient mice were euthanized and their splenocytes analyzed by flow cytometry 8 weeks after reconstitution.

### Statistical Analysis

Data were analyzed with an unpaired two-tailed Student’s t test or one-way ANOVA using the Prism software Version9 as indicated in the figure legends. Statistical significance was set at P<0.05.

## Results

### *In Vitro* Functional Screen Platform Development

**Figure 1:**
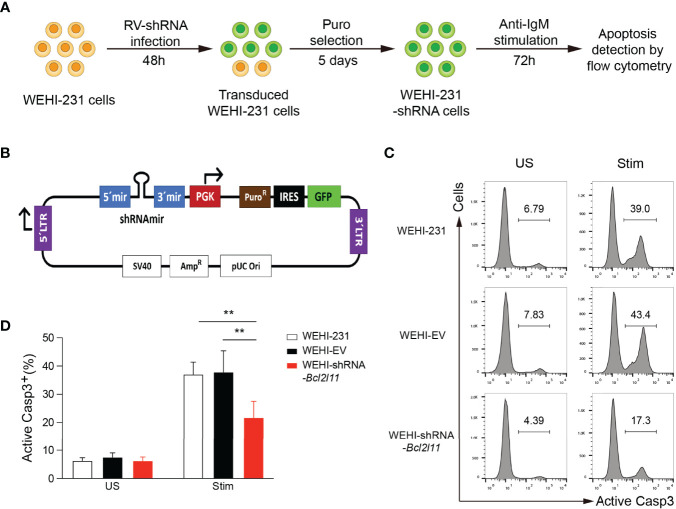
*In vitro* functional screen platform. **(A)** Screen strategy outline. WEHI-231 cells were transduced with retroviruses encoding shRNAs. After 48 hours, transduced cells were selected with puromycin for 5 days, stimulated with anti-IgM for 72 hours, stained for active caspase 3 (Casp3), and analyzed by flow cytometry. **(B)** Scheme of the TransOMIC library pMLP vector including the shRNA and puromycin selection cassettes. **(C)** Flow cytometry histograms showing apoptosis of control non-transduced WEHI-231, WEHI-EV (empty vector) and WEHI–shRNA-*Bcl2l11* cells stimulated for 72 hours with 2µg/ml anti-IgM (Stim) or left unstimulated (US), assessed by staining of active caspase 3. **(D)** Frequency of active caspase-3-positive (Casp3^+^) cells among total cells as in **(C)**. The results are representative of 4 independent experiments. Data are represented as mean + SD. Statistical analysis was performed with a one-way ANOVA test with **P < 0.01.

WEHI-231 is a commonly used immature B cell line that undergoes cell cycle arrest at G0/G1 phase and apoptosis upon anti-IgM-induced BCR crosslinking (25). This mimics the recognition of self-antigens by and subsequent clonal deletion (apoptosis) of autoreactive B cells that occur *in vivo* under homeostatic conditions. We previously found that miR-148a protects WEHI-231 cells from BCR engagement-induced apoptosis triggered by anti-IgM stimulation. This recapitulated the role of miR-148a in B cell central tolerance, where it prevented clonal deletion of autoreactive B cells following self-antigen encounter (20). We further identified 119 protein-coding genes that contained predicted miR-148a binding sites in their mRNAs and showed at least 1.5-fold reduction in their mRNA levels upon miR-148a overexpression. Four of them were chosen for experimental validation. Among them, *Bcl2l11* (encoding Bim), *Pten* and *Gadd45a* were validated as regulators of B cell tolerance, while *Tnfrsf1b* did not play any significant role in this process (20). In this study, we investigated the function of the other miR-148a target genes in B cell tolerance, first in the WEHI-231 system, followed by validation in animal models (**Figure 1A**). To this end, a retroviral TransOMIC shRNA library containing 288 shRNAs (**Supplementary Table 1**) covering 106 miR-148a target genes, with one to nine shRNAs for each gene, was generated. An empty vector (EV) and a vector encoding an shRNA against *Bcl2l11* (shRNA-*Bcl2l11*) were included as negative and positive controls. The backbone vector used to generate this library contains a shRNA cassette followed by a puromycin resistance gene that enables puromycin selection of transduced cells (**Figure 1B**). Retroviruses were produced from individual constructs to generate the shRNA retroviral library as previously described (20).

The strategy for our *in vitro* functional screen is outlined in **Figure 1A**. WEHI-231 cells were transduced with retroviruses expressing individual shRNAs. After 48 hours, transduced cells were selected with puromycin for 5 days. Half of the cells were then stimulated with anti-IgM (2μg/ml) for 72 hours to induce BCR-engagement induced apoptosis, while the other half were left without stimulation and served as control. After 72 hours of stimulation, cells were harvested, stained for active Caspase 3, and analyzed by flow cytometry to quantify the percentage of apoptotic cells as a readout for clonal deletion. WEHI-231 cells transduced with empty retroviruses (WEHI-EV) and with retroviruses encoding shRNA-*Bcl2l11* (WEHI-shRNA-*Bcl2l11*) were included in every independent experiment as controls.

The feasibility of this approach was first validated with WEHI-EV and WEHI-shRNA-*Bcl2l11* controls. As expected, a significant fraction of non-transduced and WEHI-EV-transduced WEHI-231 cells died by apoptosis when stimulated with anti-IgM, and cell death was largely abrogated in shRNA-*Bcl2l11*-transduced cells (**Figures 1C, D**). This protection from clonal deletion by decreasing the expression levels of Bim recapitulated our previous results obtained in the IgM^b^-macroself B cell tolerance mouse model (20).

### Functional Screen of the miR-148a Targetome shRNA Library

Once the *in vitro* screen platform had been validated, 288 WEHI-231 stable cell lines expressing individual shRNAs for miR-148a target genes were generated. WEHI-231 cells were transduced with retroviruses encoding individual shRNAs, selected with puromycin, stimulated with anti-IgM, and analyzed for apoptosis as described above (**Figure 1A**). The effect of shRNAs that significantly impaired BCR engagement-induced apoptosis were confirmed in three independent experiments.

**Figure 2:**
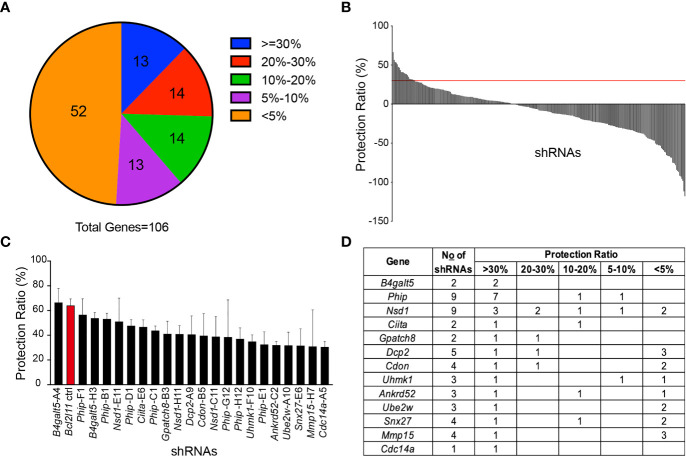
Functional screen of the TransOMIC shRNA library. **(A)** Pie chart summarizing the screen results. Numbers inside the chart indicate the number of genes with shRNAs protecting WEHI-231 cells from BCR engagement-induced apoptosis at various ratios. The protection ratio = 1 – (active caspase 3^+^ % of WEHI-shRNA)/(active caspase 3^+^ % of WEHI-EV) under stimulated conditions. **(B)** Bar graph showing protection ratios of individual shRNAs. The red line indicates the 30% protection threshold. **(C)** Bar graph detailing individual shRNAs with >30% protection ratios. **(D)** List of genes with shRNAs exhibiting >30% protection ratios. Data are representative of 2 independent experiments for *B4galt5*-H3 and *Phip*-E1, 3 for *B4galt5*-A4, *Phip*-F1, *Phip*-B1, *Nsd1*-E11, *Phip*-D1, *Ciita*-E6, *Phip*-C1, *Nsd1*-H11, *Dcp2*-A9, *Cdon*-B5, *Phip*-G12 and *Cdc14a*-A5, 4 for *Bcl2l11*, B*Gpatch8*-B3, *Nsd1*-C11, *Phip*-H12, *Uhmk1*-F10, *Ankrd52*-C2, *Ube2w*-A10 and *Snx27*-E6, and 5 for *Mmp15*-H7. Graph bars indicate mean + SD.

**Figure 3:**
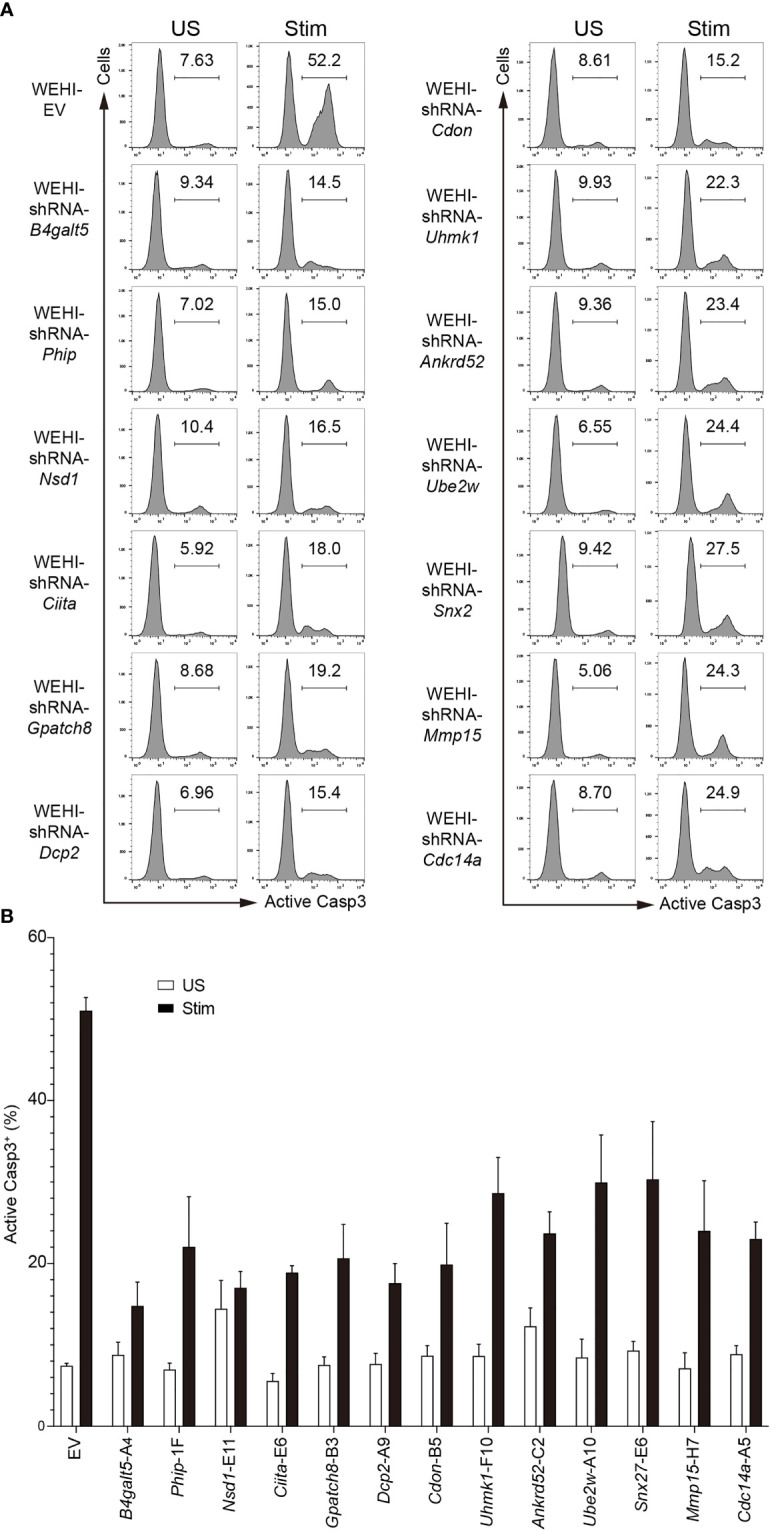
Effect of the top hit shRNAs in the *in vitro* functional screen. **(A)** Representative flow cytometry histograms showing apoptosis of WEHI-231 transduced with retroviruses encoding shRNAs or empty vector (EV), stimulated for 72 hours with 2µg/ml anti-IgM (Stim) or left unstimulated (US), followed by flow cytometry analysis of active caspase 3 (Casp3). **(B)** Frequency of active caspase-3-positive (Casp3^+^) cells among total cells in **(A)**. Every independent experiment included an internal WEHI-EV control. A representative WEHI-EV control experiment is shown. Results are pooled from 3 independent experiments for EV, *Phip*-F1, *Nsd1*-E11, *Ciita*-E6, *Dcp2-*A9, *Cdon*-B5 and *Cdc14a*-A5, 4 for *Gpatch8*-B3, *Uhmk1*-F10, *Ankrd52*-C2, *Ube2w*-A10 and *Snx27*-E6, and 5 for *B4galt5*-A4 and *Mmp15*-H7. Graph bars indicate mean + SD.

Among the 106 miR-148a target genes tested, nearly half of them showed an effect in protecting WEHI-231 cells from BCR-induced apoptosis when knocked down by shRNAs, with protection ratios ranging from 5% to 65% (**Figure 2A**). Among those, 22 shRNAs, which correspond to 13 genes, showed an average protection effect greater than 30% (**Figure 2B** and **Figure 3**). Of note, knockdown of *B4galt5*, *Phip* and *Nsd1* showed the most robust protection against BCR engagement-induced apoptosis, with all shRNAs showing consistent effect (**Figures 2C, D**).

### *In Vivo* Analysis of Positive Hits in IgM^b^-Macroself Mouse Model

**Figure 4:**
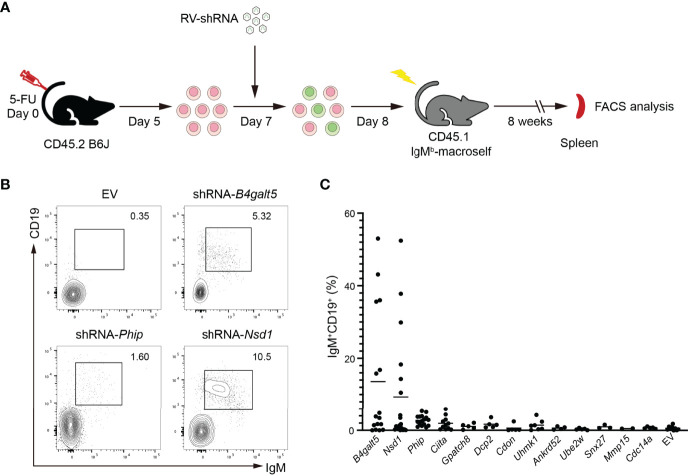
*In vivo* validation of positive hits in IgM^b^-macroself mice. **(A)** Experimental outline of the *in vivo* validation strategy. HPSCs were enriched from 5-FU-treated C57BL/6J, transduced with retroviruses encoding individual shRNAs or empty vector (EV), and used to reconstitute sublethally irradiated IgM^b^-macroself mice. After 8 weeks, the presence of splenic B cells was analyzed in these mice by flow cytometry to determine a potential break of B cell tolerance. **(B)** Representative flow cytometry plots of splenocytes from IgM^b^-macroself recipient mice reconstituted with donor HSPCs transduced with retrovirus encoding the ‘top hit’ shRNAs (*B4galt5* and *Nsd1*) or no shRNA (EV, empty vector) at terminal analysis. Numbers adjacent to outlined areas indicate percentage of IgM^+^CD19^+^ cells (B cells) among splenocytes. **(C)** Graph summarizing percentages of IgM^+^CD19^+^ splenic B cells in IgM^b^-macroself mouse model reconstituted with HPSCs transduced with top hit shRNAs. Each dot indicates an individual mouse, and the horizontal lines indicate the mean value of mice analyzed for each group with n=16 for *B4galt5*, n=19 for *Nsd1*, n=16 for *Phip*, n=11 for *Ciita*, n=7 for *Uhmk1*, n=5 for *Gpatch8*, *Dcp2*, *Cdon*,*Ube2w* and *Cdc14a*, n=4 for *Ankrd52*, n=3 for *Snx27*, n=2 for *Mmp15*, and n=9 for EV. No statistically significant differences were found.

We next investigated the function of those positive hits in regulating B cell tolerance *in vivo*, by retroviral transduction of HSPCs and reconstitution of IgM^b^-macroself mice. Briefly, C57BL/6J mice were treated with 5-fluorouracil (5-FU) for 5 days. HSPCs from treated mice were enriched, transduced with shRNA-encoding retroviruses, and transferred to sublethally irradiated IgM^b^-macroself mice. After 8 weeks, the presence of peripheral B cells was analyzed in the spleen of these reconstituted mice by flow cytometry (**Figure 4A**). We set the presence of 5% splenic B cells as the threshold to consider an effect of breaking B cell tolerance because irradiated IgM^b^-macroself mice reconstituted with wild-type C57BL/6J bone marrow cells show some background of splenic B cells after 8 weeks, up to this percentage in some experiments.

We observed a significant number of splenic B cells in 6 out of 16 (37%) and 6 out of 19 (32%) IgM^b^-macroself mice reconstituted with HPSCs transduced with shRNA-*B4galt5* and shRNA-*Nsd1*, respectively. Of note, a combination of two different shRNAs for *B4galt5* was required for this effect, as transduction of HSPCs with individual shRNAs failed to break B cell tolerance (**Supplementary Figure 1**). The remaining mice did not show compromised B cell tolerance (**Figures 4B, C**). Although shRNA-*Phip* showed a significant protection against BCR engagement-induced apoptosis in WEHI-231 cells, those shRNAs did not show much effect in IgM^b^-macroself mice, suggesting that *Phip* does not play any significant role in regulating B cell tolerance (**Figures 4B, C**). All other hits from the screen failed to break B cell tolerance *in vivo*.

### *In Vivo* Validation by a Genetic Approach

**Figure 5:**
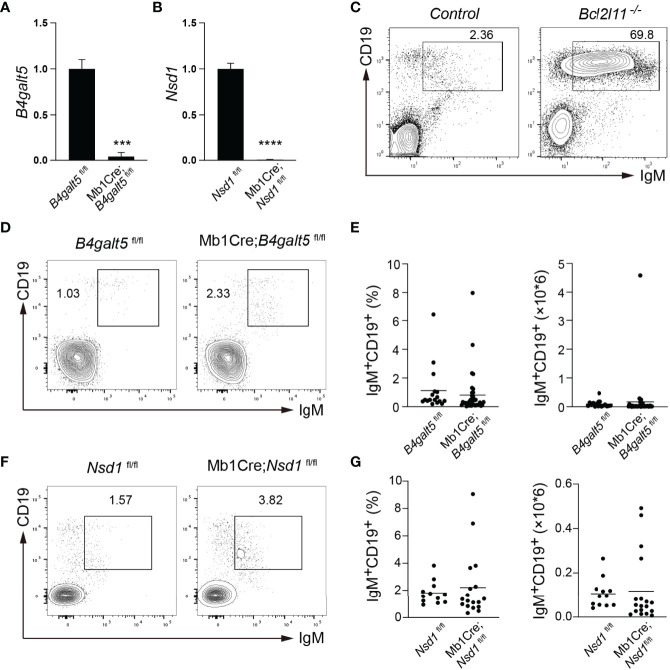
*In vivo* validation in IgM^b^-macroself mice with bone marrow cells from conditional knockout mice. **(A, B)** Expression levels of *B4galt5* **(A)** and *Nsd1* **(B)** in splenic B cells from Mb1Cre;*B4galt5*^fl/fl^ and Mb1Cre;*Nsd1*^fl/fl^ mice analyzed by qRT-PCR. Bar graph shows mean + SD. Data are representative of 2 independent experiments using technical triplicates. **(C)** Representative flow cytometry plots analyzing splenocytes from IgM^b^-macroself mice reconstituted with bone marrow cells from C57BL/6J mice and *Bcl2l11*^-/-^ mice at terminal analysis. Numbers adjacent to outlined areas indicate percentage of IgM^+^CD19^+^ B cells among splenocytes. **(D)** Representative flow cytometry plots analyzing splenocytes from IgM^b^-macroself mice reconstituted with bone marrow cells from *B4galt5*^fl/fl^ and Mb1Cre;*B4galt5*^fl/fl^ mice at terminal analysis. Numbers adjacent to outlined areas indicate percentage of IgM^+^CD19^+^ B cells among splenocytes. **(E)** Graphs summarizing percentages and numbers of IgM^+^CD19^+^ splenic B cells of mice analyzed in **(D)**. **(F)** Representative flow cytometry analysis of splenocytes from IgM^b^-macroself mice reconstituted with bone marrow cells from *Nsd1*^fl/fl^ and Mb1Cre;*Nsd*^fl/fl^ mice at terminal analysis, with numbers adjacent to outlined areas indicating percentage of IgM^+^CD19^+^ B cells among splenocytes. **(G)** Graphs summarizing percentages and numbers of IgM^+^CD19^+^ splenic B cells of mice analyzed in **(D)**. Data in **(E)** and **(G)** are pooled from 3 and 2 independent experiments, respectively with n=17 for *B4galt5*^fl/fl^, n=41 for Mb1Cre;*B4galt5*^fl/fl^, n=12 for *Nsd1*^fl/fl^, and n=18 for Mb1Cre;*Nsd1*^fl/fl^ mice. Data in **(C)** are adapted from (20). Statistical analysis was performed with an unpaired two-tailed Student’s T-test with ***P < 0.001 and ****P < 0.0001. No statistically significant differences were found in **(D, F)**.

To confirm that *B4galt5* and *Nsd1* indeed regulate B cell tolerance, we obtained conditional knockout (KO) mice with specific deletion of these genes in B cells. *B4galt5*^fl/+^ and *Nsd1*^fl/+^ mice were bred with Mb1Cre mice to drive specific deletion in the B cell lineage at the early pro-B cell stage. Deletion of these genes was validated in the resulting Mb1Cre;*B4galt5*^fl/fl^ and Mb1Cre;*Nsd1*^fl/fl^ mice by qRT-PCR analysis of splenic B cells (**Figures 5A, B**). Bone marrow cells from Mb1Cre;*B4galt5*^fl/fl^ and Mb1Cre;*Nsd1*^fl/fl^ mice were used to reconstitute irradiated IgM^b^-macroself mice. Splenic B cells of recipient mice were analyzed 8 weeks after reconstitution. No accumulation of splenic B cells was observed in IgM^b^-macroself mice reconstituted with bone marrow cells from Mb1Cre;*B4galt5*^fl/fl^, Mb1Cre;*Nsd1*^fl/fl^ or their littermate controls *B4galt5*^fl/fl^ and *Nsd1*^fl/fl^ at terminal analysis (**Figures 5C, G**). Concurrently, bone marrow cells from heterozygous Mb1Cre;*B4galt5*^fl/+^ also failed to break B cell tolerance in IgM^b^-macroself mice (**Supplementary Figure 2**). This indicates that B4galt5 and Nsd1 do not play significant roles in the regulation of B cell tolerance. Taken together, our previous and this studies show that *Bcl2l11*, *Pten* and *Gadd45a* play key roles in mediating miR-148a-regulation of B cell central tolerance, with minimal, if any, contribution from the other target genes (20).

## Discussion

B cell tolerance mechanisms are essential for the generation of a vast pool of B cells that enable the defense against a large variety of pathogens without attacking self-tissues. When B cell tolerance checkpoints are compromised, the development of autoimmune diseases ensue (1, 6, 20, 26–33). Aiming to advance our knowledge on B cell tolerance, we investigated the function of more than one hundred miR-148a target genes in this process.

Our previous study showed that elevated expression of miR-148a impaired BCR engagement-induced apoptosis in WEHI-231 cells, and that this was recapitulated in IgM^b^-macroself mice, suggesting that WEHI-231 cells are a good *in vitro* model for studying B cell central tolerance. In this study, we performed initial screen of miR-148a target genes in WEHI-231 cells and identified 13 genes whose knockdown protected BCR engagement-induced apoptosis by more than 30%. While this murine immature B cell line have proven useful to advance our understanding of B cell tolerance, it does not fully recapitulate the behavior of primary immature B cells in mice, in which cells interact with other cell subsets from their environment such as bone marrow stromal cells. Further validation of the screen hits by retroviral transduction and bone marrow reconstitution of IgM^b^-macroself mice showed that shRNA-mediated knockdown of two of those 13 genes, *B4galt5* or *Nsd1*, led to break of B cell tolerance in a fraction of IgM^b^-macroself mice. When irradiated IgM^b^-macroself mice were reconstituted with bone marrow cells from mutant mice with B cell-specific deletion of *B4galt5* or *Nsd1*, very few B cells escaped B cell central tolerance. Therefore, none of those 13 genes whose knockdown protected BCR engagement-induced apoptosis in WEHI-231 cells plays important roles in regulating B cell central tolerance. We speculate that the escape of B cell tolerance in some of the IgM^b^-macroself mice reconstituted with HPSCs transduced with retroviruses encoding shRNAs for *B4galt5* or *Nsd1* was due to off-target effect of these shRNAs.

MiRNAs bind target gene mRNAs mainly through pairing with their seed regions, allowing for the regulation of hundreds of target genes by one miRNA. Exactly how many targets mediate the function of a miRNA has been hotly debated. Cellular context-dependence of miRNA functions adds another layer of complexity to this issue (16, 18, 34, 35). For example, miR-148a regulates antigen presentation of TLR-triggered dendritic cells (36), osteoclastogenesis of CD14^+^ peripheral blood mononuclear cells (37), LDL receptor activity in human hepatic cells (38), and functions as a tumor suppressor miRNA in various types of cancers including gastric, colorectal, hepatocellular and pancreatic cancers (39–42). How the same miRNA exerts such diverse functions in different cell types remains elusive. It is possible that these differences rely on the differential gene expression programs of each individual cell type and that miRNA-mediated regulation of target genes is only efficient when target genes are expressed at appropriate levels in the cell type of interest. Another possibility is that only a small number of the hundreds of target genes regulated by a given miRNA are both functionally important and sensitive to changes in their cellular concentrations in any given cell type. Those genes would be the key target genes mediating the function of this miRNA in this particular cellular context. In the context of B cell central tolerance, three key target genes (i.e. *Bcl2l11*, *Pten* and *Gadd45a*) drive the function of miR-148a upon engagement of their BCRs with anti-IgM, with a minor contribution, if any, from the other one hundred or so target genes. Further exploring if miR-148a functions similarly or not in B cells receiving different stimuli such as TLR agonist or anti-CD40 and anti-IgM would be of interest (21, 43–45).

Our results support the concept that miRNAs exert their regulatory functions through a limited number of key target genes. In future studies, it would be interesting to perform functional analysis of miR-148a target genes in other cellular contexts to find out whether the same or different sets of key target genes mediate the function of this miRNA in different types of cells.

## Data Availability Statement

The raw data supporting the conclusions of this article will be made available by the authors, without undue reservation.

## Ethics Statement

The animal study was reviewed and approved by Animal Care and Use Committees of Xiamen University and Autonoma University of Madrid.

## Author Contributions

AG-M and CX designed and supervised the project. FM performed most of the experiments, analyzed the data and prepared the figures. YZ, RB-C, WY, AG-M, and MA performed some experiments. ZH and RB-C helped with data analysis and manuscript writing. AG-M, FM, ZH, and CX wrote the manuscript. All authors contributed to the article and approved the submitted version.

## Funding

This work was supported by the grants RTI2018-100008-A-I00 funded by Spanish Ministry of Science and Innovation MCIN/AEI/10.13039/501100011033/and FEDER, SI1-PJI-2019-00241 from Community of Madrid (Spain), merit award RyC-21155 funded by Spanish Ministry of Science and Innovation MCIN/AEI/10.13039/501100011033 and FSE to AG-M, and by the National Natural Science Foundation of China (81830047 to CX).

## Conflict of Interest

The authors declare that the research was conducted in the absence of any commercial or financial relationships that could be construed as a potential conflict of interest.

## Publisher’s Note

All claims expressed in this article are solely those of the authors and do not necessarily represent those of their affiliated organizations, or those of the publisher, the editors and the reviewers. Any product that may be evaluated in this article, or claim that may be made by its manufacturer, is not guaranteed or endorsed by the publisher.

## Supplementary Material

Supplementary Table 1 List of the shRNA sequences encoded by the TransOMIC-shRNA library.

Supplementary Figure 1 Retroviral transduction of HSPCs with individual shRNAs for B4galt5 does not induce a break of B cell tolerance. **(A)** Representative flow cytometry plots showing IgM^+^CD19^+^ splenic B cells of IgMb-macroself mice reconstituted with HSPCs transduced with an empty retrovirus, retroviruses encoding shRNA-*B4galt5*-H3, shRNA-*B4galt5*-A4 or a combination of both shRNAs (H3+A4), as indicated, 8 weeks after reconstitution. The total amount of viral particles was kept constant in all conditions. **(B)** Graph summarizing percentage of IgM^+^CD19^+^ splenic B cells among total cells of mice analyzed in **(A)**. Data in **(B)** are pooled from 4 independent experiments with n=16 for shRNA-*B4galt5*-(H3+A4), n=3 for shRNA-*B4galt5*-(H3+A4), n=4 for shRNA-*B4Galt5*-A4, and n=9 for EV. No statistical differences were found.

Supplementary Figure 2 Heterozigous deletion of *B4Galt5* does not compromise B cell tolerance. **(A)** Representative flow cytometry plots showing IgM+CD19+ splenic B cells of IgMb-macroself mice reconstituted with bone marrow cells from *B4galt5*^fl/fl^ and Mb1Cre;*B4galt5*^fl/+^ mice, as indicated, 8 weeks after reconstitution. **(B)** Graph showing percentage of IgM^+^CD19^+^ splenic B cells among total cells in mice analyzed in **(A)**. n=4 for *B4galt5*^fl/fl^ and n=6 for Mb1Cre;B4galt5fl/+ mice. No statistical differences were found.
