# Supplementary figures and images for: Analysis of a miR-148a Targetome in B Cell Central Tolerance
Source: Front Immunol. 2022 May 12;13:861655. doi: 10.3389/fimmu.2022.861655 (PMC9134011; doi:10.3389/fimmu.2022.861655)

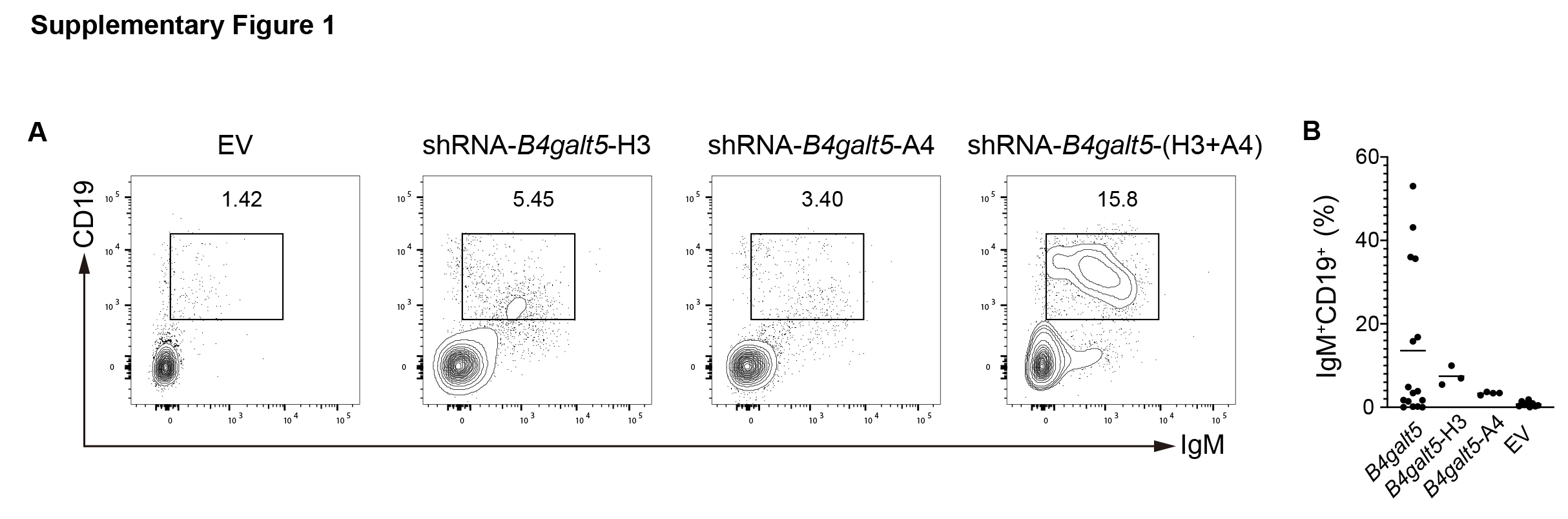

Supplement: Supplementary Figure 1 — Retroviral transduction of HSPCs with individual shRNAs for B4galt5 does not induce a break of B cell tolerance. (A) Representative flow cytometry plots showing IgM+CD19+ splenic B cells of IgMb-macroself mice reconstituted with HSPCs transduced with an empty retrovirus, retroviruses encoding shRNA-B4galt5-H3, shRNA-B4galt5-A4 or a combination of both shRNAs (H3+A4), as indicated, 8 weeks after reconstitution. The total amount of viral particles was kept constant in all conditions. (B) Graph summarizing percentage of IgM+CD19+ splenic B cells among total cells of mice analyzed in (A). Data in (B) are pooled from 4 independent experiments with n=16 for shRNA-B4galt5-(H3+A4), n=3 for shRNA-B4galt5-(H3+A4), n=4 for shRNA-B4Galt5-A4, and n=9 for EV. No statistical differences were found. [file Image_1.jpeg]

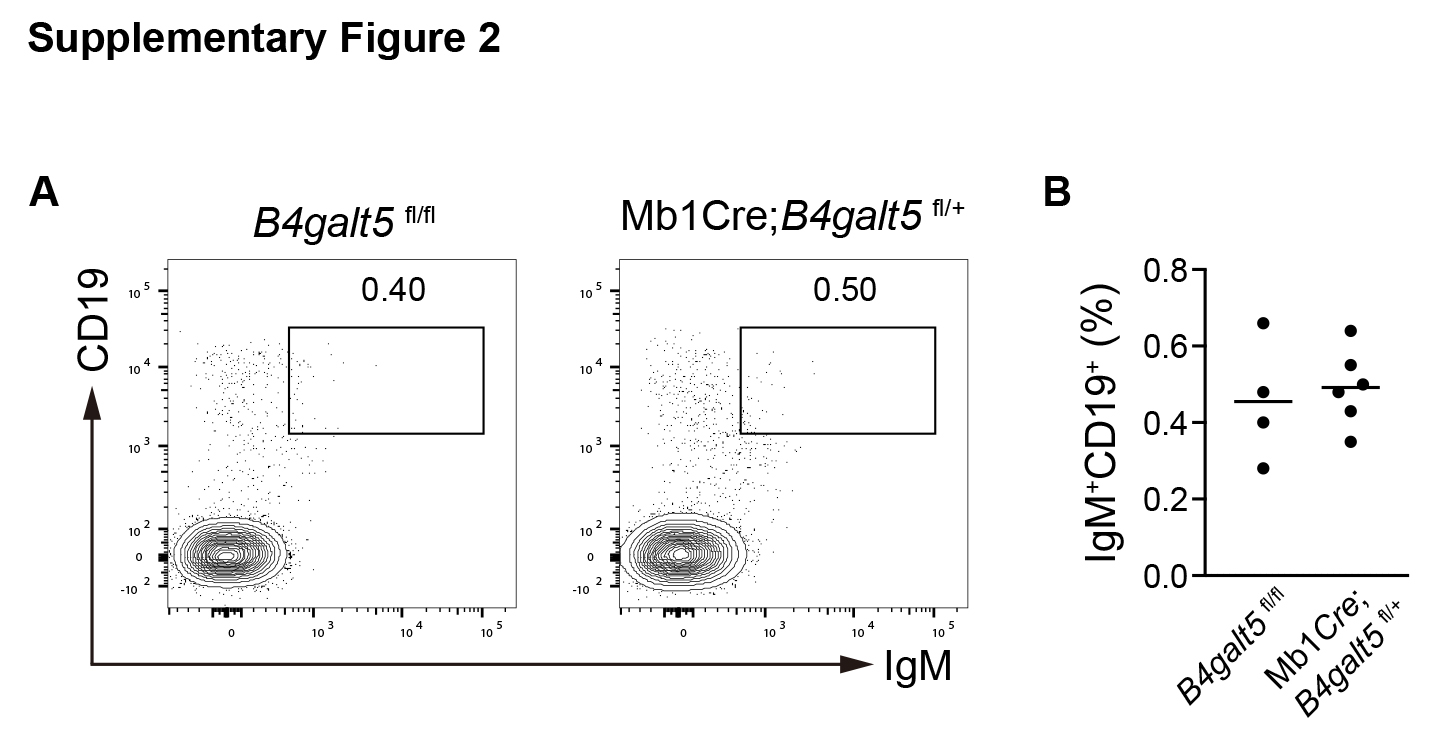

Supplement: Supplementary Figure 2 — Heterozigous deletion of B4Galt5 does not compromise B cell tolerance. (A) Representative flow cytometry plots showing IgM+CD19+ splenic B cells of IgMb-macroself mice reconstituted with bone marrow cells from B4galt5fl/fl and Mb1Cre;B4galt5fl/+ mice, as indicated, 8 weeks after reconstitution. (B) Graph showing percentage of IgM+CD19+ splenic B cells among total cells in mice analyzed in (A). n=4 for B4galt5fl/fl and n=6 for Mb1Cre;B4galt5fl/+ mice. No statistical differences were found. [file Image_2.jpeg]
